# Resolving METTL5 Specificity: Direct RNA Sequencing Reveals No Compelling Evidence for METTL5 mediated mRNA m6A Methylation in mESCs

**DOI:** 10.17912/micropub.biology.002169

**Published:** 2026-05-08

**Authors:** Samuel Le Cam, Magdalena Valenta, Robert Schneider

**Affiliations:** 1 IFE, Helmholtz München, Neuherberg, BY, DE

## Abstract

The methyltransferase-like 5 (METTL5) protein has been shown to catalyze m6A deposition on 18S ribosomal RNA. However, whether it also methylates mRNAs remains unclear. To address this, we employed direct RNA sequencing (ONT) for m6A detection on native mRNA molecules. We first validated the quantitative detection of m6A by ONT by treating mESCs with a METTL3 inhibitor. We then compared methylation levels of m6A sites between *Mettl5* -KO and WT mESCs. Our analysis provides no compelling evidence for METTL5 mediated mRNA methylation in vivo, indicating that its catalytic activity is restricted to rRNA.

**Figure 1. m6A profiling reveals no pronounced m6A loss upon Mettl5 deletion:** (A) Schematic representing ONT pipeline for m6A calling and experimental design. (B) Methylation levels (%) of individual m6A sites detected by ONT direct sequencing of mRNAs extracted from STM2457 treated and untreated (DMSO control) mESCs. Methylation levels (%) represent the number of reads with called m6A/total number of reads covering a given site. Red dots: m6A sites not responsive to STM2457 treatment. (C) Venn diagram of methylated sites (≥ 25%) identified in the two *Mettl5* -KO clones. (D) Methylation levels (%) of individual m6A sites detected on mRNAs from *Mettl5* -KO versus WT mESCs. Mean methylation values of two *Mettl5* -KO clones are plotted. (E) log2FC of m6A methylation levels at individual sites in the two *Mettl5* -KO clones (X and Y axis) compared to WT. Sites with increased or reduced m6A levels in both *Mettl5* -KO clones are highlighted in green and red, respectively. (B, D, E) Pearson correlation coefficients (R) are indicated. (F) Methylation levels of m6A mRNA sites in WT mESCs. M6A sites with reduced methylation levels in both *Mettl5* -KO clones are highlighted in red. Cut-off: 25% methylation. (G) log2FC of mean m6A load per transcript in the two *Mettl5* -KO clones over WT. Transcripts containing at least one reduced m6A site are highlighted in yellow. Transcripts with m6A load changes are highlighted in red. (H) Tracks of m6A levels (%) at *Smc4* exon 19, detected by ONT direct RNA sequencing. Bottom track: RefSeq gene annotation.

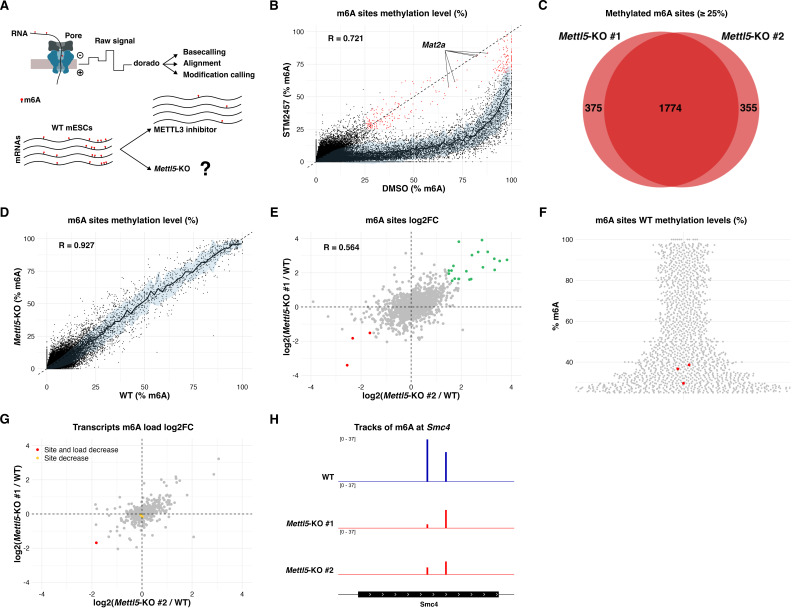



## Description

RNAs can be post-transcriptionally modified. Such modifications regulate all aspects of their function, including splicing, sub-cellular localization, stability, translation and interactions within ribonucleoprotein complexes (Motorin & Helm, 2011; Roundtree et al., 2017). Among those modifications, N6-methyladenosine (m6A) is the most abundant internal mRNA modification, present on a large set of transcripts, often on multiple sites. It has been suggested that the overall level of m6A on a given mRNA, referred to as m6A load, determines its functional impact, particularly on RNA half-life (Schwartz et al., 2014; Uzonyi et al., 2023). M6A deposition on mRNA is primarily catalyzed by METTL3 in complex with METTL14 and WTAP on DRACH motifs (D = A/G/U, R = A/G, H = A/C/U) (Liu et al., 2014; Ping et al., 2014; Wang et al., 2014). However, other m6A-depositing enzymes with more restricted substrate specificity have been identified. For example, ZCCHC4 methylates 28S rRNA on A4220 (Ma et al., 2019), while METTL16 targets U6 snRNAs and *Mat2a* mRNA at conserved hairpins, contributing to the regulation of S-Adenosyl Methionine (SAM) homeostasis (Pendleton et al., 2017). We and others have previously shown that METTL5 methylates 18S rRNA on A1832 (Ignatova et al., 2020; Leismann et al., 2020; Rong et al., 2020; Sepich-Poore et al., 2022; van Tran et al., 2019). This site is located in the decoding center of the ribosome, suggesting a role in fine-tuning translation (Rong et al., 2020).

However, it remains unclear whether these enzymes have additional targets, particularly on mRNA. For METTL5, such activity had been suggested *in vitro* (e.g. Ignatova et al., 2020). A major challenge in addressing this question is the limited quantitative nature and resolution of antibody-based methods such as methylated RNA immunoprecipitation (meRIP) which is widely used for transcriptome-wide m6A profiling (Sepich-Poore et al., 2022). To investigate the effects of *Mettl5* loss on m6A in mRNA, we used direct RNA sequencing by Oxford Nanopore Technologies (ONT) which allows quantitative m6A calling with single-nucleotide resolution on native mRNA molecules ( Figure 1A ).

We first assessed the accuracy and quantitative performance of ONT direct RNA sequencing for m6A detection. For this, we treated mouse embryonic stem cells (mESCs) with the METTL3 inhibitor STM2457 (Yankova et al., 2021). We then sequenced mRNAs from STM2457-treated and DMSO-treated mESCs on a single flow cell using adaptor-barcoding for multiplexing (Van Der Toorn et al., 2025). Methylation calling was performed using the Dorado basecaller without restriction to DRACH motifs, to maximize the number of detected m6A sites. Sites were defined as methylated if covered by least 25 reads and with a methylation level above 25%. As expected, STM2457 treatment resulted in a strong decrease (mean=71.4%) in the modification level of m6A sites above threshold ( Figure 1B ). Only 20.4% of the methylated sites present in the control sample were detected upon METTL3 inhibition. Virtually all sites called as methylated in the treated condition were detectable in the control. Together, these data validate quantitative m6A detection by ONT direct RNA sequencing. Interestingly, 283 sites were not responsive to METTL3 inhibition ( Figure 1B ), suggesting a potential m6A deposition by other methyltransferases. Among them were the well characterized METTL16 target sites on *Mat2a* ( Figure 1B ). The unchanged methylation level of these m6A sites upon treatment could also reflect a high stability of the transcripts harboring them. However, in most cases, the same transcripts also contained m6A sites with decreased m6A levels.

Having the experimental setup established, we next performed ONT sequencing of mRNAs extracted from two independent *Mettl5* knockout (KO) mESC clones (Ignatova et al., 2020). Overall, detected m6A sites were highly overlapping between the two *Mettl5* -KO clones ( Figure 1C ) and their methylation levels were highly similar to their wild-type (WT) counterpart with a Pearson correlation of 0.918 ( Figure 1D ). This is consistent with the majority of m6A on mRNAs being deposited by METTL3. We thus focused next on convergent site-specific methylation changes (log2FC) shared between both *Mettl5* -KO clones, relative to the WT. Overall, changes in methylation levels of m6A sites were correlated between the two KO mESC clones ( Figure 1E ). Among the 2002 identified m6A sites, 21 showed an increase in methylation (log2FC ≥ 1.5) in both Mettl5-KO clones, likely due to indirect effects of *Mettl5* deletion. Interestingly, only three m6A sites showed decreased m6A levels in both *Mettl5* -KO clones (log2FC ≤ -1.5) and were thus potential candidates for METTL5-dependent m6A sites. We found that these three candidate sites have, compared to all methylated m6A sites, relatively low methylation levels ( Figure 1F ). This low methylation stoichiometry warrants cautious interpretation, as methylation level changes at such m6A sites is more susceptible to variability.

Consistent with this, these three putative m6A sites did not pass the detection threshold in the METTL3 inhibition dataset. We next calculated methylation levels per transcript (m6a loads), as this metric reflects transcript-wide methylation levels. We detected a decrease of methylation load only for one RNA in METTL5 KOs: *Smc4* , while other candidate sites were on transcripts with unchanged overall m6A loads ( Figure 1G, H), indicating their limited impact on RNA function. However, as the two m6A sites in *Smc4* were not detected in the METTL3 inhibition dataset, they might reflect experimental or technical variability rather than bona fide METTL5-dependent mRNA methylation.

Taken together, we detected upon Mettl5 depletion in mESCs only very few and low confidence decreases in m6A levels on mRNAs. The three putative Mettl5-dependent m6A sites identified with reduced methylation levels upon Mettl5 depletion could be attributable to limited detection robustness at low stoichiometry, as these sites are below detection thresholds in independent dataset. Of note these findings are based on a single cell type and two knock out clones and may therefore reflect - or fail to cover - context-specific effects. Our analysis focuses on highly expressed transcripts and is subjected to the known limitations of direct RNA sequencing including biases towards shorter transcripts and 3'-end coverage. In summary, our quantitative m6A profiling provides little supporting evidence for a transcriptome-wide role of METTL5 in mRNA methylation, thereby helping to resolve a key open question regarding METTL5 substrate specificity in vivo.

## Methods

Cell lines:

For the control dataset, E14 mESCs with following modifications were used : 2C::3xtbGFP-PEST; Zscan4::mCherry-PEST; Dox::APOBEC1-YTH-HA-IRES-tdiRFP (non-induced); METTL3- FKBP12 ^F36V^ : homozygous knock-in of FKBP12 ^F36V^ in Mettl3 C-terminus (non-induced).

For *Mettl5* deletion, the cell lines used were generated in a previous study (Ignatova et al., 2020).

Cell culture:

Mouse embryonic stem cells (mESCs) were cultured on gelatin-coated dishes in Serum + LIF medium consisting of Dulbecco’s Modified Eagle Medium (DMEM) supplemented with 15 % fetal bovine serum (FBS, PAN-Biotech), 0.1 μM β-mercaptoethanol (Gibco), 2 mM L-glutamine (Gibco), 1 × non-essential amino acids (Gibco), 100 U/ml penicillin, 100 µg/ml streptomycin (Gibco) and recombinant Leukemia inhibitory factor (LIF; produced by IGBMC ESC facility). For maintenance of cells, the medium was supplemented with 2i (1 μM PD032591 and 3 μM CHIR99021, Axon). Cells were passed with accutase (Sigma-Aldrich) every 2 days. For the chemical inhibition of METTL3 catalytic activity, cells were treated with 30 μM STM2457 (Biomol) for 48h. Control cells were treated with 0.025% v/v DMSO.

Nanopore direct RNA sequencing:

Total RNA was isolated using TRIzol-Chloroform extraction (Thermo Fisher Scientific) followed by iso-propanol precipitation, according to the manufacturer’s protocol. Total RNA was ligated to poly-T WarpDemuX RTA adapters for multiplexing (Van Der Toorn et al., 2025) using T4 DNA ligase (NEB) and reverse-transcribed to form RNA-cDNA hybrid using the SuperScript III Reverse Transcriptase (Thermo Fisher Scientific). The end of library preparation was performed using the SQK-RNA004 kit (ONT) according to the manufacturer’s instructions. Libraries were loaded onto FLO-MIN004RA flow cells. For the validation dataset, a total of 1.28 and 1.05 million pass reads were obtained with a median read length of 562bp and 523bp for DMSO and STM2457 conditions, respectively. For the *Mettl5* -KO dataset, a total of 0.68, 0.41 and 0.51 million pass reads were obtained with a median read length of 481bp, 614bp, 518bp for the WT, *Mettl5* -KO #1 and *Mettl5* -KO #2, respectively.

Data processing:

Raw pod5 files generated by ONT direct RNA sequencing were demultiplexed using WarpDemuX (Van Der Toorn et al., 2025). Basecalling, modification calling and alignment to the mouse genome (mm10) were performed using dorado (dorado-1.4.0, rna004_130bps_sup@v5.3.0, inosine_m6A_2OmeA@v1). Modification summary counts bed files were generated using modkit pileup (v0.4.4) and were then restricted to m6A sites with a coverage above 25 reads within each sample, for all samples of a given dataset. Sites that were not overlapping with an exon (UCSC RefSeq) were filtered out using bedtools intersectBed. Bedgraph files with methylation level information were generated from these filtered summary counts bed files and restricted to m6A sites above 25% in at least one sample within each dataset. These thresholds are applied to ensure a maximal robustness of calculated methylation levels but also limit the analysis to transcripts with enough expression and to sites closer to the 3’ end of RNA as sequencing is primed from the polyA tail. For m6A loads, the mean value per transcript was calculated across all sites that fall within the aforementioned thresholds.

Data availability:

Data are available under the GEO accession GSE328291.

## Data Availability

Description: Density distribution of m6A sites in WT mESCs as a function of methylation level as an alternative view of Figure 1F without cut-off. Red lines indicate m6A sites with reduced methylation levels in both Mettl5-KO clones. 25% methylation level is indicated by a dotted line.. Resource Type: Image. DOI: https://doi.org/10.22002/ww9vz-47694

## References

[R1] Motorin Yuri, Helm Mark (2011). RNA nucleotide methylation. WIREs RNA.

[R2] Roundtree Ian A., Evans Molly E., Pan Tao, He Chuan (2017). Dynamic RNA Modifications in Gene Expression Regulation. Cell.

[R3] Schwartz Schraga, Mumbach Maxwell R., Jovanovic Marko, Wang Tim, Maciag Karolina, Bushkin G. Guy, Mertins Philipp, Ter-Ovanesyan Dmitry, Habib Naomi, Cacchiarelli Davide, Sanjana Neville E., Freinkman Elizaveta, Pacold Michael E., Satija Rahul, Mikkelsen Tarjei S., Hacohen Nir, Zhang Feng, Carr Steven A., Lander Eric S., Regev Aviv (2014). Perturbation of m6A Writers Reveals Two Distinct Classes of mRNA Methylation at Internal and 5′ Sites. Cell Reports.

[R4] Uzonyi Anna, Dierks David, Nir Ronit, Kwon Oh Sung, Toth Ursula, Barbosa Isabelle, Burel Cindy, Brandis Alexander, Rossmanith Walter, Le Hir Hervé, Slobodin Boris, Schwartz Schraga (2023). Exclusion of m6A from splice-site proximal regions by the exon junction complex dictates m6A topologies and mRNA stability. Molecular Cell.

[R5] Liu Jianzhao, Yue Yanan, Han Dali, Wang Xiao, Fu Ye, Zhang Liang, Jia Guifang, Yu Miao, Lu Zhike, Deng Xin, Dai Qing, Chen Weizhong, He Chuan (2013). A METTL3–METTL14 complex mediates mammalian nuclear RNA N6-adenosine methylation. Nature Chemical Biology.

[R6] Ping Xiao-Li, Sun Bao-Fa, Wang Lu, Xiao Wen, Yang Xin, Wang Wen-Jia, Adhikari Samir, Shi Yue, Lv Ying, Chen Yu-Sheng, Zhao Xu, Li Ang, Yang Ying, Dahal Ujwal, Lou Xiao-Min, Liu Xi, Huang Jun, Yuan Wei-Ping, Zhu Xiao-Fan, Cheng Tao, Zhao Yong-Liang, Wang Xinquan, Danielsen Jannie M Rendtlew, Liu Feng, Yang Yun-Gui (2014). Mammalian WTAP is a regulatory subunit of the RNA N6-methyladenosine methyltransferase. Cell Research.

[R7] Wang Yang, Li Yue, Toth Julia I., Petroski Matthew D., Zhang Zhaolei, Zhao Jing Crystal (2014). N6-methyladenosine modification destabilizes developmental regulators in embryonic stem cells. Nature Cell Biology.

[R8] Ma Honghui, Wang Xiaoyun, Cai Jiabin, Dai Qing, Natchiar S. Kundhavai, Lv Ruitu, Chen Kai, Lu Zhike, Chen Hao, Shi Yujiang Geno, Lan Fei, Fan Jia, Klaholz Bruno P., Pan Tao, Shi Yang, He Chuan (2018). N6-Methyladenosine methyltransferase ZCCHC4 mediates ribosomal RNA methylation. Nature Chemical Biology.

[R9] Pendleton Kathryn E., Chen Beibei, Liu Kuanqing, Hunter Olga V., Xie Yang, Tu Benjamin P., Conrad Nicholas K. (2017). The U6 snRNA m 6 A Methyltransferase METTL16 Regulates SAM Synthetase Intron Retention. Cell.

[R10] van Tran Nhan, Ernst Felix G M, Hawley Ben R, Zorbas Christiane, Ulryck Nathalie, Hackert Philipp, Bohnsack Katherine E, Bohnsack Markus T, Jaffrey Samie R, Graille Marc, Lafontaine Denis L J (2019). The human 18S rRNA m6A methyltransferase METTL5 is stabilized by TRMT112. Nucleic Acids Research.

[R11] Ignatova Valentina V., Stolz Paul, Kaiser Steffen, Gustafsson Tobias H., Lastres Palma Rico, Sanz-Moreno Adrián, Cho Yi-Li, Amarie Oana V., Aguilar-Pimentel Antonio, Klein-Rodewald Tanja, Calzada-Wack Julia, Becker Lore, Marschall Susan, Kraiger Markus, Garrett Lillian, Seisenberger Claudia, Hölter Sabine M., Borland Kayla, Van De Logt Erik, Jansen Pascal W.T.C., Baltissen Marijke P., Valenta Magdalena, Vermeulen Michiel, Wurst Wolfgang, Gailus-Durner Valerie, Fuchs Helmut, Hrabe de Angelis Martin, Rando Oliver J., Kellner Stefanie M., Bultmann Sebastian, Schneider Robert (2020). The rRNA m
^6^
A methyltransferase METTL5 is involved in pluripotency and developmental programs. Genes & Development.

[R12] Leismann Jessica, Spagnuolo Mariangela, Pradhan Mihika, Wacheul Ludivine, Vu Minh Anh, Musheev Michael, Mier Pablo, Andrade‐Navarro Miguel A, Graille Marc, Niehrs Christof, Lafontaine Denis LJ, Roignant Jean‐Yves (2020). The 18S ribosomal RNAm
^6^
A methyltransferase Mettl5 is required for normal walking behavior in
*Drosophila*. EMBO reports.

[R13] Rong Bowen, Zhang Qian, Wan Jinkai, Xing Shenghui, Dai Ruofei, Li Yuan, Cai Jiabin, Xie Jiaying, Song Yang, Chen Jiawei, Zhang Lei, Yan Guoquan, Zhang Wen, Gao Hai, Han Jing-Dong J., Qu Qianhui, Ma Honghui, Tian Ye, Lan Fei (2020). Ribosome 18S m6A Methyltransferase METTL5 Promotes Translation Initiation and Breast Cancer Cell Growth. Cell Reports.

[R14] Sepich-Poore Caraline, Zheng Zhong, Schmitt Emily, Wen Kailong, Zhang Zijie Scott, Cui Xiao-Long, Dai Qing, Zhu Allen C., Zhang Linda, Sanchez Castillo Arantxa, Tan Haiyan, Peng Junmin, Zhuang Xiaoxi, He Chuan, Nachtergaele Sigrid (2022). The METTL5-TRMT112 N6-methyladenosine methyltransferase complex regulates mRNA translation via 18S rRNA methylation. Journal of Biological Chemistry.

[R15] Yankova Eliza, Blackaby Wesley, Albertella Mark, Rak Justyna, De Braekeleer Etienne, Tsagkogeorga Georgia, Pilka Ewa S., Aspris Demetrios, Leggate Dan, Hendrick Alan G., Webster Natalie A., Andrews Byron, Fosbeary Richard, Guest Patrick, Irigoyen Nerea, Eleftheriou Maria, Gozdecka Malgorzata, Dias Joao M. L., Bannister Andrew J., Vick Binje, Jeremias Irmela, Vassiliou George S., Rausch Oliver, Tzelepis Konstantinos, Kouzarides Tony (2021). Small-molecule inhibition of METTL3 as a strategy against myeloid leukaemia. Nature.

[R16] van der Toorn Wiep, Bohn Patrick, Liu-Wei Wang, Olguin-Nava Marco, Gribling-Burrer Anne-Sophie, Smyth Redmond P., von Kleist Max (2025). Demultiplexing and barcode-specific adaptive sampling for nanopore direct RNA sequencing. Nature Communications.

